# Sex-Biased Dispersal at Different Geographical Scales in a Cooperative Breeder from Fragmented Rainforest

**DOI:** 10.1371/journal.pone.0071624

**Published:** 2013-08-09

**Authors:** Carl Vangestel, Tom Callens, Viki Vandomme, Luc Lens

**Affiliations:** Terrestrial Ecology Unit, Ghent University, Ghent, Belgium; Aarhus University, Denmark

## Abstract

Dispersal affects both social behavior and population structure and is therefore a key determinant of long-term population persistence. However, dispersal strategies and responses to spatial habitat alteration may differ between sexes. Here we analyzed spatial and temporal variation in ten polymorphic microsatellite DNA loci of male and female Cabanis’s greenbuls ( *Phyllastrephus* *cabanisi* ), a cooperative breeder of Afrotropical rainforest, to quantify rates of gene flow and fine-grained genetic structuring within and among fragmented populations. We found genetic evidence for female-biased dispersal at small spatial scales, but not at the landscape level. Local autocorrelation analysis provided evidence of positive genetic structure within 300 m distance ranges, which is consistent with behavioral observations of short-distance natal dispersal. At a landscape scale, individual-based autocorrelation values decreased over time while levels of admixture increased, possibly indicating increased gene flow over the past decade.

## Introduction

Dispersal plays a major role in contemporary dynamics and the evolutionary potential of a population [1]. However, current connectivity (i.e., resistance experienced by individuals to move from one location to another) - and concomitant dispersal behavior - is often heavily affected by anthropogenic habitat change, particularly in tropical ecosystems [2]. The escalating rate at which tropical rainforest is being fragmented and degraded imposes severe constraints on historical trajectories of genes and individuals [3,4], thereby threatening the long-term persistence of numerous rainforest species through loss of genetic diversity and lack of resilience against stochastic perturbations [5–8]. Moreover, empirical evidence has accumulated that species exhibiting asymmetrical dispersal behavior, i.e., when one sex is more dispersive than the other, are even more prone to having distorted population dynamics in response to environmental variation [9–13]. Assembling detailed knowledge of sex-biased dispersal patterns is therefore critical in formulating appropriate species-specific conservation schemes.

In many bird species, particularly those with a complex breeding system such as cooperative breeders, each sex faces different selective pressures and consequently shows dissimilar dispersal strategies. According to the resource hypothesis [14], female-biased dispersal predominates in birds as male birds are the most active sex in both resource and territory defense and hence benefit more from being familiar with their natal surrounding [14,15]. Male offspring therefore often reside in their natal territory assisting their parents’ reproduction efforts at the cost of delaying their own breeding attempts, but with the prospects of filling up nearby breeding territories that become vacant in the future [14,16]. Recent findings suggest that the extent of differential gene flow between sexes may be scale dependent. At a larger scale, between-fragment dispersal may be heavily influenced by the intervening matrix in such a way that selective forces are invariant with respect to both sexes, ultimately balancing dispersal rates in females and males at a regional scale [17]. Obtaining direct empirical evidence of sex-biased dispersal through capture-recapture studies remains a cumbersome and laborious undertaking. Alternatively, the availability and decreasing cost of polymorphic genetic markers has made it possible to indirectly infer the prevalence and extent of such patterns, as asymmetric dispersal typically results in higher genetic similarity between neighbors and more genetic structure in the philopatric sex [18–20].

Genetic methods used to infer sex-biased dispersal can broadly be classified into three categories, i.e., models based on Wright’s F-statistics [21], assignment tests that identify the most likely origin of a genotype, and models that assess the relationship between geographic and genetic distance such as spatial autocorrelations [19]. F-statistics are performed at the level of ‘populations’. They rather reflect historical gene flow and are often based on inappropriate assumptions (see 22 for a review), while *a priori* designation of population units may often lead to doubtful estimates of genetic structure [23–25]. Alongside methodological improvements on the analysis of F-based statistics, statistical models have been developed to quantify dispersal by assigning individuals to their most likely population of origin, with operational units being “individual genotypes” rather than populations [16]. Despite the irrefutable advantages of such an approach, these analyses do not provide information on local (i.e., within-population) levels of gene flow without invoking artificial sub-structuring [26]. Spatial autocorrelation analysis does not suffer from these constraints and evidence is growing that this analytical approach appears to be the most powerful method for detecting subtle signatures of sex-biased dispersal [16].

Here we apply all three methods to study both coarse-grained and fine-grained patterns of spatial genetic structure and sex-biased dispersal in a cooperatively breeding forest bird (Cabanis’s greenbul; *Phyllastrephus* *cabanisi* ). Multivariate spatial autocorrelation previously revealed fine-grained genetic patterning in a variety of species [16,20,27–32], and is increasingly used in a conservation context ( [33] for a recent example). Signatures of sex-biased dispersal are further evaluated using population structure and assignment metrics. Cabanis’s greenbul commonly breeds in the interior of rainforest fragments in south-east Kenya where it maintains a (facultative) cooperative breeding strategy [34]. Such a strategy may potentially foster non-random genetic patterns at small spatial scales. To date, such patterns may have remained cryptic as earlier genetic studies on this species were performed at the population-level only [34,35]. By comparing levels of past population differentiation (estimated from microsatellite genotypes) with contemporary dispersal rates (estimated from multi-strata capture–recapture models), Callens et al. [35] showed loss of mobility over time. In contrast, spatiotemporal genetic and demographic analyses over a much shorter time span showed increased, rather than decreased, population levels of genetic variation, possibly reflecting a recent increase in genetic connectivity (i.e., gene flow among fragments) [34]. The amount of knowledge accumulated about such regional patterns and processes sharply contrasts with the paucity of data on either within-fragment genetic structure or sex-biased dispersal patterns. Using a set of ten polymorphic microsatellite DNA loci, we here study individual-based genetic distance measures at different spatial scales, testing the following hypotheses: (i) genetic and geographical distance matrices co-vary at a local (i.e., within fragments) and regional scale (i.e., among fragments); (ii) the strength of these associations decreases over a recent, fifteen year time span ; and (iii) sex-biased dispersal occurs at a local scale but not at the regional level.

## Material and Methods

### Ethics Statement

This study was conducted under research permits NCST/5/002/R/274/4 and NCST/RRI/12/1/BS-011/58 of the Kenyan National Council for Science and Technology and research permit EC2012-052 of the Ethical Committee of Ghent University, Belgium. Permission to work in the study area was granted by the Taita Taveta District Commissioner, while all field procedures complied with Belgian and Kenyan guidelines on animal welfare. All necessary steps were taken to minimize animal suffering during handling, and birds were never kept in captivity or injured by any means.

### Study area and species

**Figure 1:**
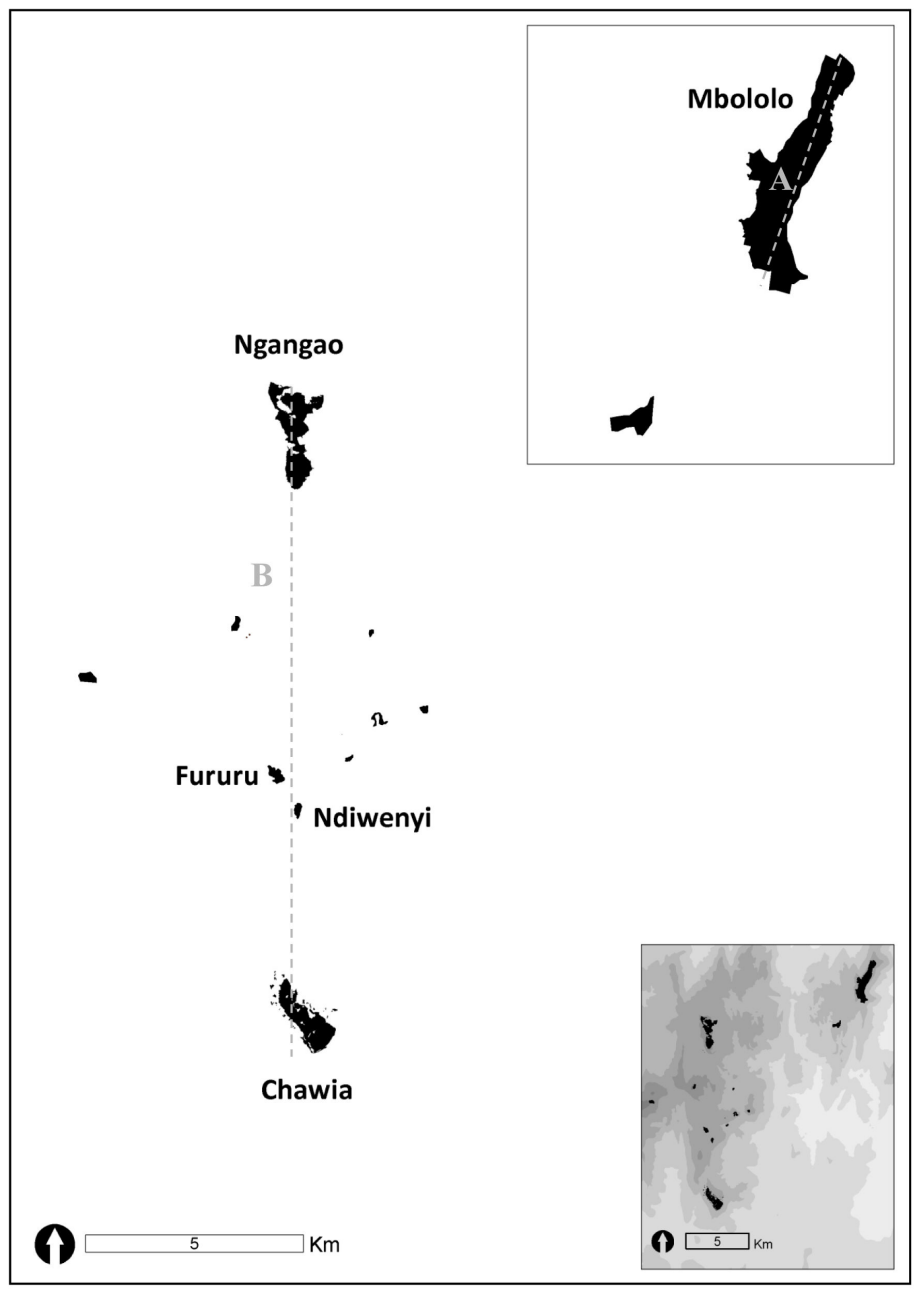
Map of the study area with the location of three large (MB, CH, NG) and two small (FU, ND) indigenous forest fragments that hold breeding populations of cabanis’s greenbul ( *Phyllastrephus* *cabanisi* ). Analyses were performed along linear transects at two different geographical scales: a local (within-fragment) scale in fragment MB (A), and a landscape (among-fragment) scale including fragments CH, NG, FU and ND (B).

The Taita Hills (SE Kenya, 03°24’ S, 38°21’ E) represent the northernmost extreme of the Eastern Arc Mountains, a chain of mountains that run from south-eastern Kenya to southern Tanzania [36]. They boast a high diversity of flora and fauna with high levels of endemism, but are also faced with high levels of deforestation [37–39]. Forest loss within the Eastern Arc Mountains has been both rapid and drastic and indigenous forests are currently scattered over ca. 5076 km^2^ [40]. Within the Taita Hills, the total area of indigenous forest cover decreased by ca. 50% between 1955 and 2004 [41], mainly due to clearance for small-subsistence agriculture [42,43]. At present, the Taita forest archipelago covers 430 ha of indigenous forest fragmented into three larger patches (between 86 and 185 ha) and eight tiny remnants (2-8 ha), located on two mountain isolates (Dabida and Mbololo) separated by a low-altitude valley (Paranga) [41,44,45] (Figure 1).

*P* *. cabanisi* is a medium-sized passerine of ‘least conservation concern’ [46] that inhabits central- to east-African moist forest [47,48]. The species displays facultative cooperative breeding behavior, with individuals living in small family groups that consist of the adult breeding pair and up to three helpers [34]. Within the Taita forest archipelago, the species has been recorded in all 11 indigenous forest remnants, but has only been found breeding in the three largest fragments (Mbololo (MB) 185 ha, Ngangao (NG) 120 ha, Chawia (CH) 86 ha) and two small ones (Fururu (FU) 8 ha, Ndiwenyi (without date) 4 ha) (Figure 1: fragments NG, CH, FU and ND are located on a single mountain isolate). In this study, sampling was restricted to breeding populations only, whereby the spatial configuration of fragmentation allowed us to apply a one-dimensional (linear) sampling design (i.e., both within and among fragments). For within-fragment analysis, individuals were extensively sampled along a 3 km linear transect in fragment MB (see Figure 1A). For among-fragment analysis, individuals were sampled in four isolated forest fragments (NG, FU, ND, CH) positioned along a 13.5 km linear transect across a matrix of small subsistence agriculture (see Figure 1B). Fragment MB was excluded from this latter analysis as the Paranga valley running between the Mbololo and Dabida mountains constrains dispersal to and from this population as indicated by strong genetic divergence between MB and the other fragments [34,35].

### Post-fledging dispersal

Between 1996 and 2012, a total of 1436 different individuals were trapped in mist-nets of which 48% were recaptured at least once. Upon capture, birds were aged (following [49]), banded, and released at the original site of capture. For all individuals first captured as “nestling”, “juvenile” or “first-year”, the exact age was known during all subsequent recaptures. For all individuals first captured as “fully grown” (>1 year old), the minimum age was known. Upon first capture of an individual, 2-3 µl of blood was collected from the brachial vein and stored in a 95% ethanol buffer. Mist-net lines were operated in one to seven 4-ha plots per fragment (proportional to fragment size) and were evenly spaced out in order to sample entire plots. Net positions, net lengths (120 m/plot) and daily trapping efforts (06-18h) were kept constant among trapping sessions. As dispersal distances based on adult capture-recaptures may be biased if fledglings emigrate from their natal territory before first capture (e.g., [50]), post-fledging dispersal distances of individuals ringed as nestlings (2007-2012) were quantified by calculating Euclidian distances between the locations of their nest and the furthest recapture record. Trapping effort was equal in consecutive years, and distances were calculated using Arcmap v 9.2.

### DNA extraction, PCR and genotyping

We genotyped two subsets of individuals that were first captured during one of two time periods of equal length: first period (1996-2000), second period (2006-2010). To avoid that temporal samples would differ in signature of dispersal because of unequal representation of young individuals, only birds aged >1 year upon first capture were included in the genetic analysis. Resulting sample sizes were as follows: NG=23F/42M, CH=28F/36M, FU=9F/5M, ND=10F/7M; second period (2006-2010): MB=18F/35M, NG=22F/32M, CH=35F/20M, FU=14F/10M, ND = 8F/8M. Individuals sampled during the first period and still alive during the second one, were included in the first period only. DNA was isolated by boiling in a 5% Chelex solution (Biorad) after an incubation period of 90 min at 55° C in the presence of 100 µg proteinase K [51]. PCR protocols, product size ranges, and optimal reaction conditions of ten variable microsatellite markers are summarized in [35]. Birds were sexed molecularly using a set of sex-linked primers P2/P8 [52]. PCR products were visualized on an ABI3130 sequencer (Applied Biosystems) and microsatellite lengths were determined with GENEMAPPER 4.1. We used MICROCHECKER 2.2.3 to identify scoring errors that could be attributed to stuttering, differential amplification of size-variant alleles causing large allele drop-out or presence of null alleles [53]. Running 10000 Monte Carlo simulations and calculating 95% confidence did not provide evidence for null alleles in any of the loci under study. Deviations from Hardy–Weinberg equilibrium and linkage disequilibrium per locus were tested with GENEPOP 4.0 [54,55].

### Statistical analyses

#### Population structure

We applied a Bayesian clustering algorithm as implemented in the software program STRUCTURE to assess the number of distinct genetic clusters and level of admixture in each period separately [23]. We discarded the first 100000 MCMC iterations as a burn-in and ran three independent runs of K=1-5 for 200000 MCMC repetitions under the correlated allele frequency model. The modal value of the ad hoc quantity ΔK, based on the second order rate of change of the likelihood function was used as the criterion to infer the optimal number of genetic clusters [56].

We assessed fine- and course-grained spatial patterns of genetic structure by quantifying the association between matrices of pairwise spatial and genetic distances through spatial autocorrelation analysis [20,57,58]. Analyses were conducted using GENALEX version 6.41 [59]. We defined multiple distance class sizes (‘MultipleDclass’) as such an analysis provides a correlogram which visualizes the extent of spatial autocorrelation as a function of cumulative geographical distance. We defined multiple distance size plots to overcome the problem of sampling scheme dependency [58], and distance classes at which autocorrelation coefficients no longer remained significant were considered to approximate the true extent of identifiable genetic structure (all analyses were run for 9999 bootstraps) [20]. Boundaries of the multiple distance classes at a regional scale were chosen in such a way they resembled between-fragment distances, while at a local scale we rather aimed at obtaining equal increments in sample size per distance class.

#### Sex-biased dispersal

We used three different approaches to assess the extent of female-biased dispersal. First, we performed spatial autocorrelation analysis at a local (no GPS locations of captures available in fragment MB during the first period) and landscape scale (both sampling periods) and assessed similarities between male and female correlograms.

Second, we used four statistical descriptors as implemented in FSTAT 2.9.3.2: F_IS_, F_ST_, mean (mAI_c_) and variance (vAI_c_) of the corrected assignment index AI [60,61]. When dispersal is strongly sex biased, samples of the dispersive sex are predicted to contain a mixture of resident and immigrant individuals which causes a larger heterozygote deficit (Wahlund effect) and more positive F_IS_ values. Allelic frequencies of the philopatric sex are more strongly driven by genetic drift than by gene flow and are hence predicted to show higher levels of genetic differentiation (F_ST_) compared to those of the dispersive sex. Assignment indices reflect the probability that a multilocus genotype occurs in the sampled population, and the most dispersive sex is predicted to show lower mAI_c_ values (resident birds tend to have higher AI_c_ values than dispersers) and higher vAI_c_ values (the dispersing sex is expected to contain an assembly of both residents and dispersers). Significance and null distributions of all four statistics were assessed using 10000 permutations.

Third, we performed analyses of molecular variance (AMOVA) for each sex separately, i.e., to assess whether males and females showed similar patterns in genetic variation partitioning among and within fragments. Largest proportions of genetic variation attributed to the between-fragment component are expected in the most philopatric sex. Next we ran a hierarchical AMOVA on the combined male and female data set with sex as ‘region’ and fragment as ‘population’ to simultaneously estimate the differentiation among sexes and fragments. 999 random permutations were used to assess the level of significance and all AMOVA analyses were performed in GENALEX version 6.41 [59]. 

## Results

### Post-fledgling dispersal

**Figure 2:**
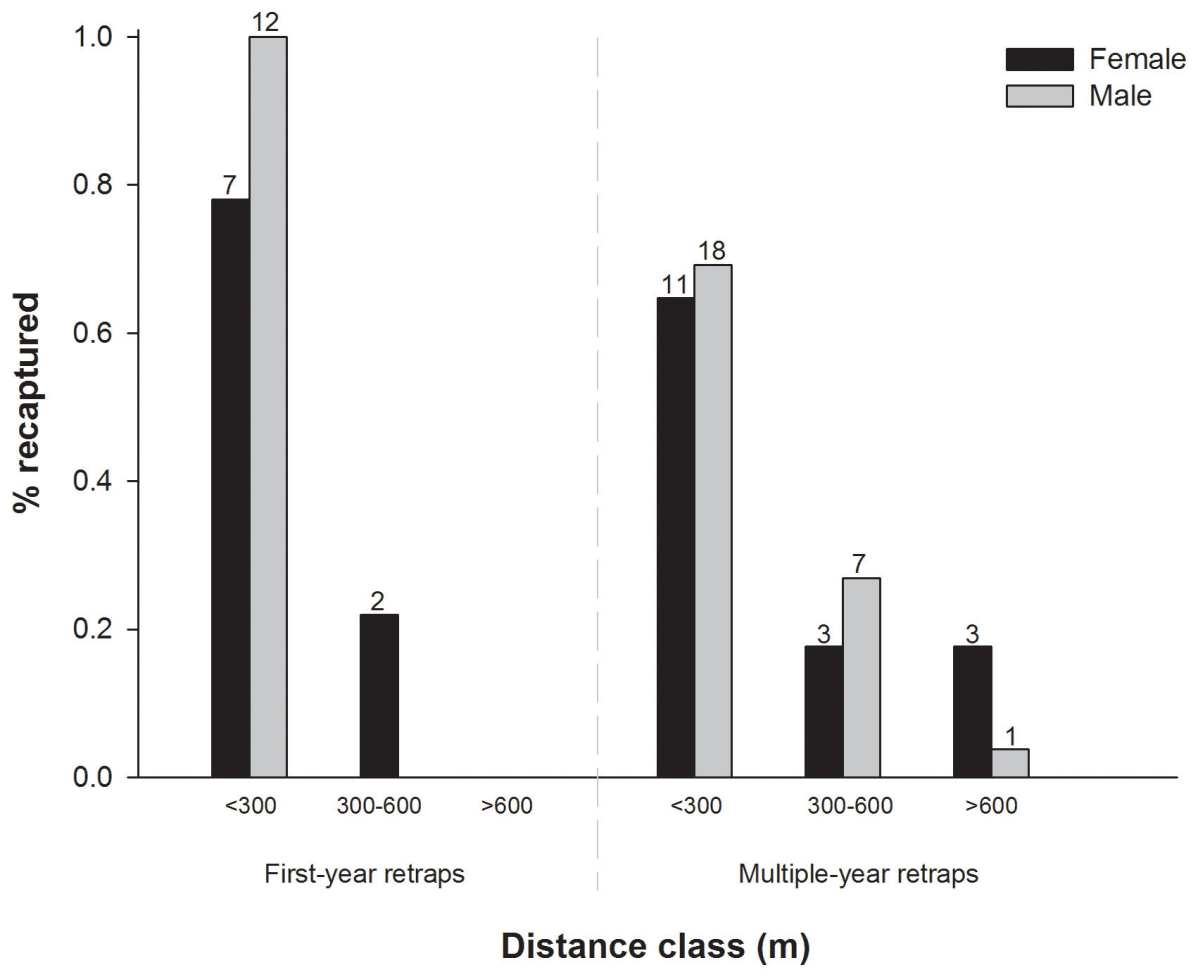
Distribution of maximum post-fledging recapture distances of *P* *. cabanisi* fledglings during their first year and over multiple years. Data were collected between 2007–2012, sample sizes are indicated above each vertical bar.

Maximum Euclidian distance analysis showed that 100% of all male fledglings were recaptured within 300 m from their natal site during their first year, while 22% of all females showed dispersal distances larger than 300m. None of the birds however dispersed further than 600m in their first year (Figure 2). When including retraps of older individuals (ringing sessions extended up to Dec 2012), only 9% (three females and one male) were recaptured more than 600 m from their natal site, two of which dispersed to a different fragment (> 4.5 km).

### Microsatellite DNA analysis

There was no evidence for scoring errors due to large allele drop-out or stutter. Loci were highly polymorphic in all populations and consistent with Hardy-Weinberg equilibrium (all *P* > 0.05 after Bonferroni correction [62]). There was no evidence for linkage disequilibrium after correction for multiple testing [62], except for the following combinations: Pca3-WBSW2 (period 1) and Pfi04-Pfl54 (period 2). Since removal of these loci did not affect our results, analyses were based on information from all loci.

### Population structure

**Figure 3:**
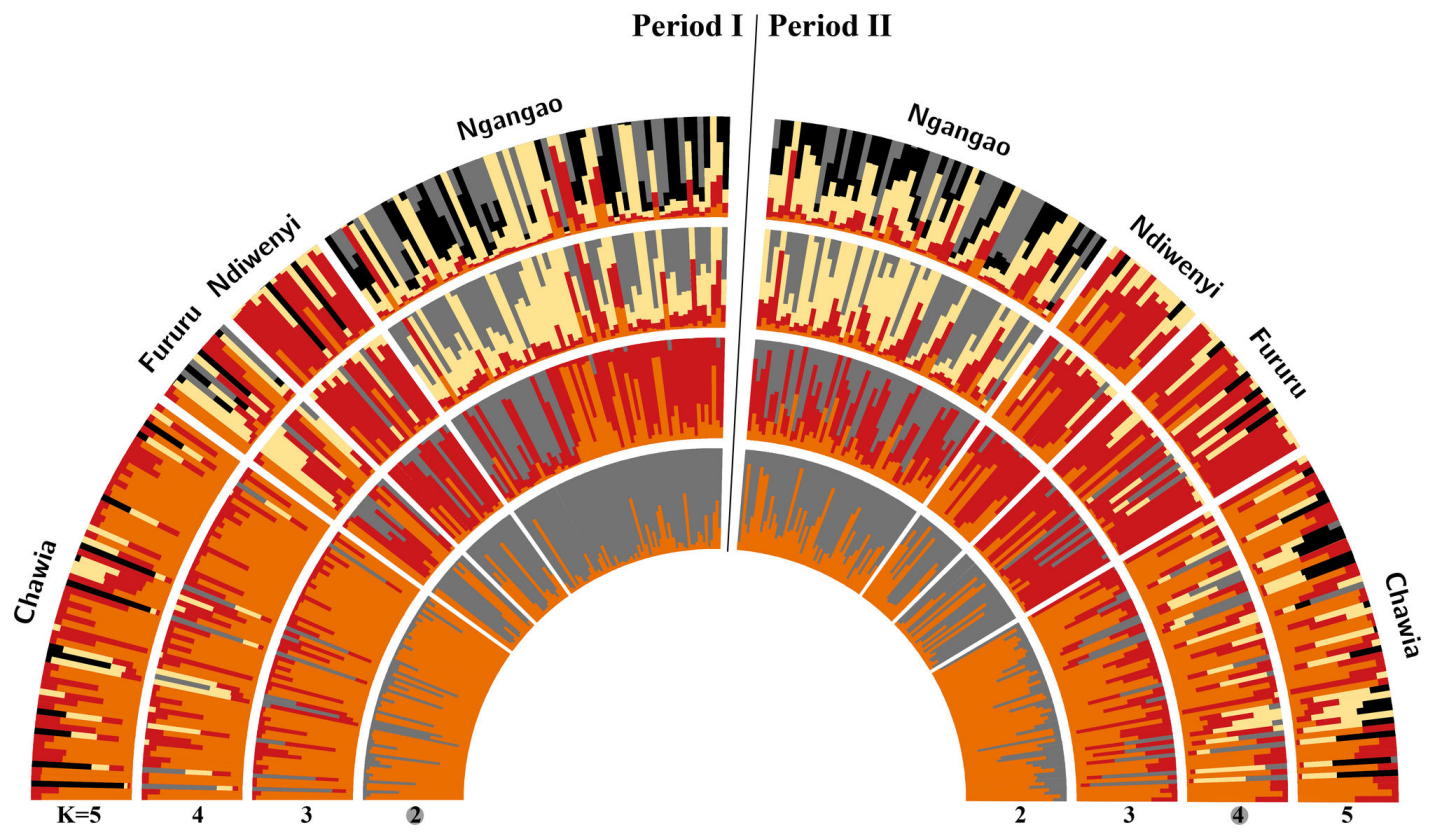
Levels of admixture provided by Bayesian clustering analyses (STRUCTURE) for each period separately (left panel: period 1/right panel: period 2). Following the procedure outlined in [56], optimal cluster sizes of K=2 and K=4 were assigned to period 1 and period 2, respectively.

We identified two genetic clusters (K=2) in period 1 (ΔK=183), whereby population CH was separated from all other populations (Table 1, Figure 3). In period 2, optimal clustering was obtained at K=4(ΔK=17), however, the majority of individuals showed no exclusive affinity to a specific cluster as inferred from the high proportion of admixed individuals. When restricting the number of clusters at the suboptimal value of K=2 (similar to period 1), the increase in admixture over time persisted.

**Table 1:** Optimal numbers of genetic clusters (K) at regional scale for period 1 and period 2, based on three independent runs of the STRUCTURE algorithm.

Period	K	Mean LnP(K)	St dev LnP(K)	Ln'(K)	|Ln''(K)|	Delta K
1	1	-4249.5000	0.1000			
	**2**	**-4111.8667**	**0.5508**	**137.6333**	**100.9667**	**183.3234**
	3	-4075.2000	20.0442	36.6667	24.9667	1.2456
	4	-4063.5000	10.0732	11.7000	47.7333	4.7386
	5	-4004.0667	13.8684	59.4333		
2	1	-4026.5333	0.3512			
	2	-3981.2000	8.2420	45.3333	25.7333	3.1222
	3	-3910.1333	9.1937	71.0667	42.8000	4.6554
	**4**	**-3881.8667**	**7.5956**	**28.2667**	**130.5000**	**17.1810**
	5	-3984.1000	5.0210	-102.2333		

For each period, optimal K-values as inferred from the Delta K method [56] are in bold.

### Sex-biased dispersal

#### Regional scale

**Figure 4:**
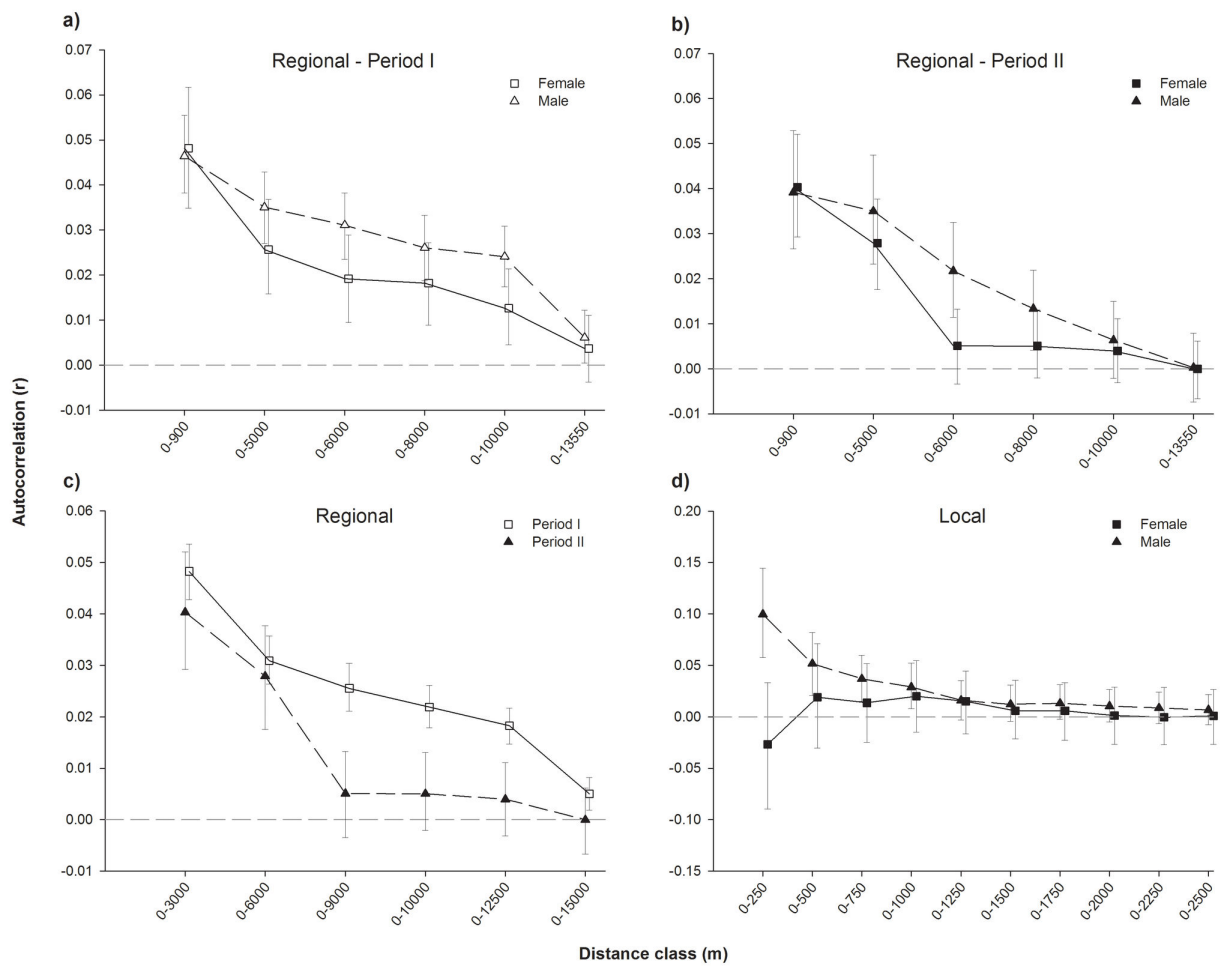
Multiple distance class plots illustrating the gradual decay of positive genetic structure with increasing geographical distance. Plots are shown for each sex at various spatio-temporal scales: at a regional scale for period 1 (a), period 2 (b) and both sexes lumped (c); at a local scale (within Mbololo) for period 2 (d). Error bars indicate 95% CI as determined by 9999 bootstraps.

**Figure 5:**
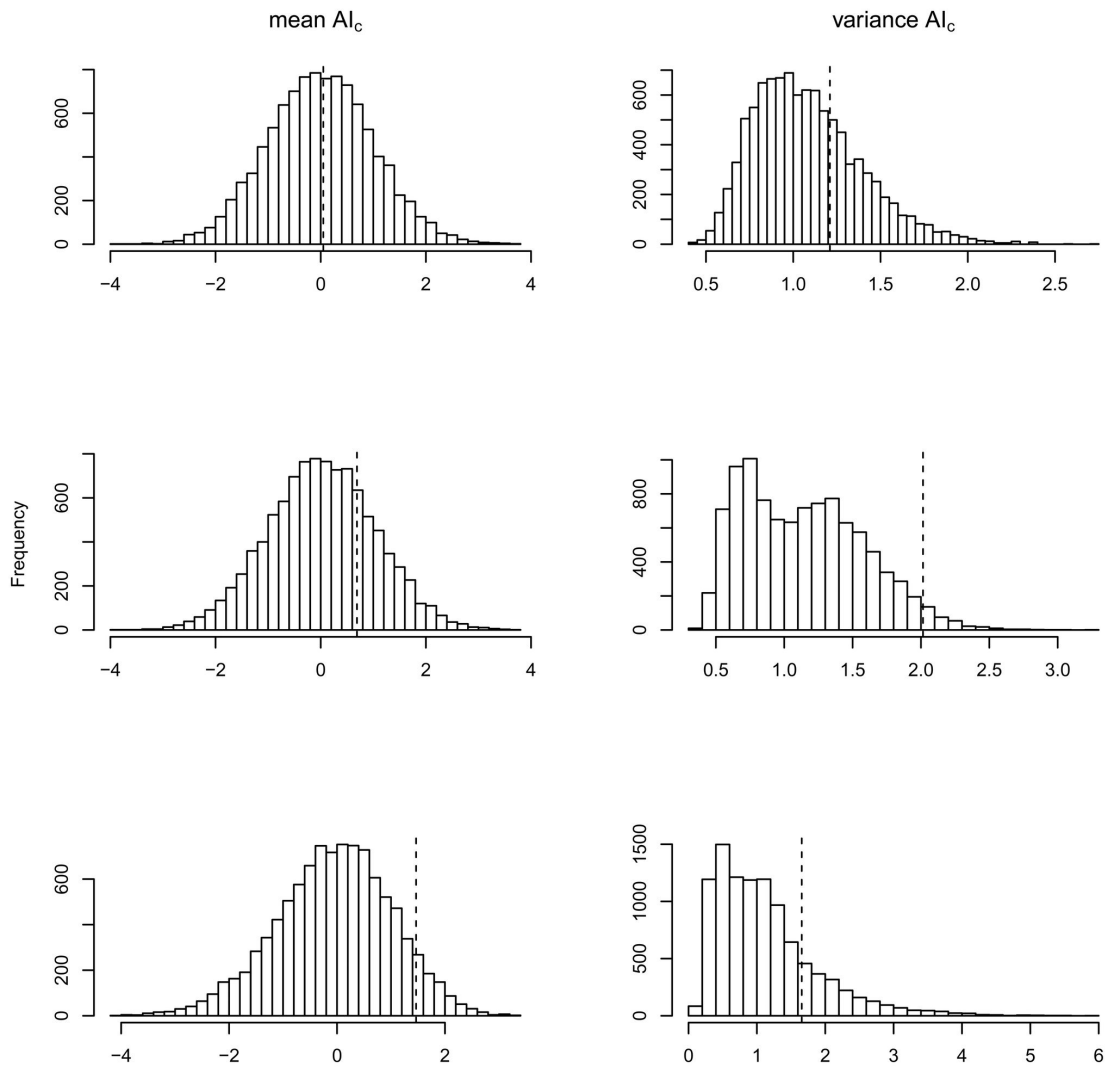
Distribution of randomized statistics under the null hypothesis of equal dispersal rates in both sexes. Permuted test statistics are ‘male mAI_c_-female mAIc’ (left) and ‘vAI_c_ female/vAI_c_ male’ (right). Observed statistics are indicated by vertical dashed line and extreme positive values indicate female-biased dispersal. Upper panel: regional - period 1; middle panel: regional - period 2; lower panel: local - period 2.

The positive genetic structure during both periods indicated a non-random spatial distribution of genotypes and a stronger genetic similarity between geographically proximate clusters. There was no strong indication of asymmetrical dispersal between sexes, although the decay of the correlation coefficient was slightly steeper for females compared to males in period 2 (Figure 4a,b). After lumping males and females, the multiple distance class correlogram showed lower genetic affinity among individuals during the second period, in particular at larger distance classes (Figure 4c). All AI metrics pointed towards female-biased dispersal, yet only vAI_c_ values were significantly higher in females in period 2 (p=0.03). Under the assumption of equal dispersal rates among sexes, a permutation-based null distribution gave rise to smaller deviations between female and male vAI_c_ values as compared to the observed difference in 97% of all cases (Figure 5, middle panel). None of the other assignment or F-based tests reached statistical significance (Table 2, Figure 5). Between-fragment differentiation in period 1 was of a comparable size when females (F_ST_= 0.077) and males (F_ST_= 0.092) were treated independently, and near-identical trends were observed in period 2 (F_ST_ females = 0.076/F_ST_ males = 0.074). Likewise, hierarchical AMOVA’s indicated similar levels of differentiation (Table 3). Hence, results of the AMOVA analysis are consistent with the lack of sex-biased dispersal at a regional scale.

**Table 2:** Sex-biased statistics obtained at two different temporal-spatial scales.

	n_f_ / n_m_	mAI_c_	vAI_c_	F_ST_	F_IS_
Regional period 1	70/90	-0.0276/0.0215 (0.534)	11.9325/9.9061 (0.779)	0.0385/0.0478 (0.712)	-0.0394/-0.0145 (0.157)
Regional period 2	79/70	-0.3242/0.3659 (0.152)	**20.9220/10.3813 (0.030)**	0.0406/0.0400 (0.444)	0.0284/0.0478 (0.679)
Local	18/35	-0.9673/0.4975 (0.099)	17.6784/10.6623 (0.187)		

‘Regional’ constitutes an among-fragment analysis while the ‘local’ analysis is restricted to a single fragment (Mbololo). Female-biased dispersal would be reflected in higher F_IS_ and vAI_C_ (but lower F_ST_ and mAI_C_) values in females compared to males, respectively. n_f_/n_m_: number of females/males; mAI_c_ and vAI_c_ refer to the median and variance of the corrected assignment indices, respectively; F_ST_/F_IS_: Wright’s (1931) fixation indices. P-values are given in parentheses and significant values (P<0.05) are indicated in bold.

**Table 3:** Genetic variation partitioning among and within fragments for each sex separately, using an AMOVA.

Period	Data	Between-sex variation (%)	Between-fragment variation (%)	F_ST_	P
Period 1	Female	-	7.7	0.077	0.01
	Male	-	9.2	0.092	0.01
Period 2	Female	-	7.6	0.076	0.01
	Male	-	7.4	0.074	0.01
Period 1	Female+male	0	8.5	0.085	0.01
Period 2	Female+male	0	7.5	0.075	0.01

In a hierarchical AMOVA (‘Female+male’ data), both sexes were analyzed simultaneously and genetic variation was portioned between sexes (‘regions’), between fragments within each sex (‘population’) and within fragments. Genetic differentiation (F_ST_) among fragments was estimated from the between-fragment component and 999 random permutations were used to assess P-values.

#### Local scale

At a local (within-fragment) scale, positive genetic structure was only observed in males, reflecting sex-biased dispersal at small spatial scale (Figure 4d). Although sex differences in mAIc and vAIc were in agreement with sex-biased dispersal no statistical significance was reached (Table 2, Figure 5).

## Discussion

Both at a local (within-fragment) and regional (between-fragment) spatial scale, Cabanis’s greenbul populations of the Taita Hills forest archipelago showed evidence of genetic sub-structuring, the extent of which gradually decreased with increasing geographical distance. Within the large forest fragment (MB), dispersal was asymmetrical between sexes, with females appearing as the most dispersive sex. At a regional scale, however, such sex bias was no longer apparent. Genetic connectivity increased over time, but there was no unequivocal evidence that this was due to a disproportional increase in gene flow by the most dispersive sex.

Patterns of dispersal are notoriously difficult to study, even when collecting large numbers of field observations or capture/recapture data over multiple years. Here we show that individual-based statistical analysis of indirect measures, such as those based on highly variable neutral genetic markers, allows inferences about sex-specific dispersal and population structure at high resolution and in a cost effective way. Results from our autocorrelation analysis support the central view that females are the most dispersive sex in birds, although this trend was not supported by subsequent assignment analysis. Because the sample size for within-fragment analysis was only moderate in our study, these results need to be interpreted cautiously. The lack of evidence for sex-biased dispersal at a regional scale, however, may be attributed to several, non-exclusive factors. First, local and regional spatial dynamics have been shown to be driven by different selection pressures. At small spatial scales, asymmetrical dispersal is generally assumed to result from selection against incestuous breeding or from differential resource acquisition [14,18,63]. Long-distance dispersal, in contrast, is associated with (re)colonizing dynamics of vacant habitat patches within a meta-population framework, and may be less prone to the evolution of sex-specific strategies [17,64]. Second, small-scale dispersal often involves movements within continuous patches of suitable habitat only, while long-distance dispersal may imply crossing of inhospitable matrices, especially in severely fragmented habitats. Gap crossing may constrain movement behavior to such an extent that natural dispersal patterns become disrupted or even reversed [12,65,66]. Third, lack of power may have prevented us from identifying asymmetrical dispersal patterns at the regional scale. Simulation studies previously showed that the statistical power of autocorrelation analysis to detect sex-dependent dispersal is highest at the spatial scale where the level of aggregation of relatives is highest [67]. Our analyses indicated strongest aggregation at a small scale (< 1km) which may have been too small for the resolution used in analyses at the regional level. Methods based on F- and AI- distributions, in turn, require intermediate dispersal rates and severely skewed dispersal rates (e.g., 20:80 rates) to detect sex-biased dispersal [18]. However, earlier studies in the Taita Hills revealed low to almost zero rates of between-fragment dispersal in Cabanis’s greenbuls and six other forest species [35,68]. Under such conditions, vAI_c_ is expected to outperform the other three methods [18], which may explain why in the second period female-biased dispersal was detected though vAI_c_ estimation only.

Autocorrelation analysis yielded fine-grained (i.e., local) spatial genetic clustering in males only, indicating that males were genetically more strongly correlated than expected by chance when separated at distances smaller than 1000-1250m. As no obvious barriers were present in fragment MB and highly mobile species such as birds are expected to be able to cross such distances without much effort, we believe that the social (breeding) structure of *P* *. cabanisi* , rather than dispersal limitation per se, is at the base of this fine-grained structuring. *P* *. cabanisi* is group-living, with adults occupying the same territory for up to 13 years (inferred from the long-term Taita ringing database). Within the study area, the species displays facultative cooperative breeding behavior with parents selecting identical nest sites during consecutive breeding cycles [34]. Albeit based on small samples, fledglings invariably settle within 600 m of their natal site during their first year of life and helper birds at the nests of adult territory owners are predominantly male offspring from the previous breeding cycle [34], (De Neve et al., unpubl. data). Based on this combined evidence, we suggest that the fine-grained genetic population structure in *P* *. cabanisi* is likely generated by philopatry and short dispersal distances, a result that is consistent with studies on other cooperatively breeding species [16,28,69]. Beyond local genetic structuring, results of this study also revealed genetic structuring at the landscape scale, likely reflecting the interplay of high philopatry and low genetic connectivity as a result of strong landscape resistance against dispersal.

Apart from spatial variation, autocorrelation analysis also revealed temporal variation, i.e., lower genetic affinity among individuals during the second sampling period, particularly at larger geographical distances (> 6000-9000 m). A decrease in genetic autocorrelation over time may reflect an increase in genetic drift and/or in long-distance gene flow [69]. An increase in genetic drift with time seems unlikely in our study, as population-level analysis previously showed that effective population sizes remained equal or slightly increased during the second period [34]. An increase in between-fragment dispersal with time, however, is consistent with the observation that (at least) two small forest remnants were colonized by *P* *. cabanisi* during the last decade [34]. Finally, a (small) decrease in population differentiation may also result in deflated autocorrelation values [67], which is consistent with the observed increase in genetic admixture over time.

In conclusion, individual-based genetic analysis revealed female-biased dispersal at a local spatial scale but failed to demonstrate a similar pattern at a larger (regional) scale. Such fine-grained genetic structuring remained undetected in earlier population-level studies on the same populations. While our analysis also suggested increased gene flow among isolated populations over time, the short time-frame involved (at least relative to the longevity of tropical birds) still hampers predictions on longer-term connectivity trends within bird populations of the Taita forest archipelago.
